# Genome-wide analysis of the laccase (LAC) gene family in *Aeluropus littoralis*: A focus on identification, evolution and expression patterns in response to abiotic stresses and ABA treatment

**DOI:** 10.3389/fpls.2023.1112354

**Published:** 2023-03-01

**Authors:** Seyyed Hamidreza Hashemipetroudi, Mozhdeh Arab, Parviz Heidari, Markus Kuhlmann

**Affiliations:** ^1^ Department of Genetic Engineering and Biology, Genetics and Agricultural Biotechnology Institute of Tabarestan (GABIT), Sari Agricultural Sciences and Natural Resources University (SANRU), Sari, Iran; ^2^ RG Heterosis, Leibniz Institute of Plant Genetics and Crop Plant Research (IPK), Gatersleben, Germany; ^3^ National Institute of Genetic Engineering and Biotechnology (NIGEB), Tehran, Iran; ^4^ Faculty of Agriculture, Shahrood University of Technology, Shahrood, Iran

**Keywords:** ABA treatment, salt stress, cold stress, gene expression, gene structure, plant gene family

## Abstract

Laccases are plant enzymes with essential functions during growth and development. These monophenoloxidases are involved in lignin polymerization, and their expression respond to environmental stress. However, studies of laccases in some plants and fungi have highlighted that many structural and functional aspects of these genes are still unknown. Here, the laccase gene family in *Aeluropus littoralis* (AlLAC) is described based on sequence structure and expression patterns under abiotic stresses and ABA treatment. Fifteen non-redundant AlLACs were identified from the *A. littoralis* genome, which showed differences in physicochemical characteristics and gene structure. Based on phylogenetic analysis, AlLACs and their orthologues were classified into five groups. A close evolutionary relationship was observed between LAC gene family members in rice and *A. littoralis*. According to the interaction network, AlLACs interact more with proteins involved in biological processes such as iron incorporation into the metallo-sulfur cluster, lignin catabolism, regulation of the symbiotic process and plant-type primary cell wall biogenesis. Gene expression analysis of selected *AlLAC*s using real-time RT (reverse transcription)-PCR revealed that *AlLAC*s are induced in response to abiotic stresses such as cold, salt, and osmotic stress, as well as ABA treatment. Moreover, *AlLAC*s showed differential expression patterns in shoot and root tissues. Our findings indicate that *AlLAC*s are preferentially involved in the late response of *A. littoralis* to abiotic stress.

## Introduction

Laccases (EC 1.10.3.2), named after their presence in the Japanese lacquer tree (*Toxicodendron vernicifluum*), are multi-copper oxidases (LMCOs). These enzymes are involved in catalyzing the oxidation of one electron of various cellular compounds such as arylamines, phenols, and aromatic thiols (Reiss et al., 2013). Members of the multi-copper oxidases superfamily include ceruloplasmin, nitrite reductase, ascorbate oxidase, and ferroxidase. Laccases have been identified in many organisms, including plants, fungi, insects, and bacteria, and are involved in a wide range of cellular processes (Wang et al., 2015a; Janusz et al., 2020; Lu et al., 2021). Plant laccases are copper-containing glycoproteins that act as key regulators for lignin polymerization and deposition in the plant cell wall (Liu et al., 2017). Laccases contain three conserved copper (Cu)-oxidase domains (Mot and Silaghi-Dumitrescu, 2012), which can be used to identify laccase family members. Due to their importance, members of this gene family have been identified and analyzed in plants, including *Lolium perenne* (Gavnholt et al., 2002), *Oryza Sativa* (Liu et al., 2017), *Sorghum bicolor* (Wang et al., 2017), *Arabidopsis thaliana* (Turlapati et al., 2011), *Brachypodium distachyon* (Wang et al., 2015b), *Zea mays* (Caparrós-Ruiz et al., 2006), *Setaria viridis* (Simões et al., 2020)*, Brassica napus* (Zhang et al., 2013), *Gossypium arboretum* (Zhang et al., 2019), *Saccharum officinarum* (Cesarino et al., 2013), *Eucalyptus grandis* (Arcuri et al., 2020) and *Pyrus bretschneideri* (Lu et al., 2021).

According to previous studies, laccases can be involved in various cellular processes. For instance, Liu and coworkers, reported that laccases are mostly induced in the early development stage of rice seedlings (Liu et al., 2017), and Simões and coworkers, showed that laccases from *Setaria viridis* are potentially involved in monolignol oxidation (Simões et al., 2020). Moreover, the study of laccases in loblolly pine revealed that these genes are expressed mainly in immature secondary xylem, the tissue for synthesized lignin (Sato et al., 2001). Lignin is critical in increasing plant vigor and resistance to biotic and abiotic stresses. Accordingly, the lignin content in the mutant line for laccases genes, *LAC17* and *LAC14*, in *Arabidopsis* was decreased compared to wild-type lines (Berthet et al., 2011), suggesting that laccases are involved in lignin polymerization and decomposition. Also the involvement of laccases in Cleome seed coat lignification was described (Zhuo et al., 2022). Other roles have also been suggested for laccases in plants, highlighting that the members of this gene family are involved in responding to environmental stresses. For instance, the overexpression of *OsChI1*, a laccase gene from rice, sharply increased the salt and drought tolerance in transgenic *Arabidopsis* (Cho et al., 2014). In addition, copper tolerance was improved in *Arabidopsis* transgenic lines for the overexpressed *OsLAC10*, a *laccase10* gene from rice (Liu et al., 2017). Similarly, Cai and coworkers reported that the *laccase2* gene in *Arabidopsis* is involved in response to abiotic stresses such as drought (Cai et al., 2006), and Xu et al. showed that laccase genes are induced by abiotic stresses such as low/high temperature, drought, and hormone application (ABA, MeJA, and SA) in citrus (Xu et al., 2019). Based on their broad substrate spectrum and ability to depolymerize lignin, laccases moved into the focus of current research. Furthermore they became of economic importance (Riva, 2006; Arregui et al., 2019) due to their use as bioactive component of ascorbic acid (vitamin C) sensors (Lee et al., 2018).

*Aeluropus littoralis* is a halophyte monocot model that can grow under salt and drought conditions (Saad et al., 2011; Hashemi et al., 2016). *A. littoralis* does not have the ability to be cultivated, but as a valuable source of resistance genes, it can be used in breeding and genetic engineering programs. The genomic resources for *A. littoralis* provide the opportunity to identify the key genes and molecular mechanisms of response to abiotic stresses (Hashemi et al., 2016). Motivated by the importance of laccase genes in increasing the resistance of plants against environmental conditions, the present study analyzed the sequence structure of laccase genes as well as their regulatory systems. In addition, the expression pattern of these genes in response to environmental stress and ABA hormone was investigated in root and shoot tissues.

## Materials and methods

### Identification of laccase genes

The protein sequences of *Arabidopsis thaliana* laccase family genes (17 members) were retrieved from the *Arabidopsis* information resource TAIR10 (TAIRv10, http://www.arabidopsis.org/). The sequences were cross-checked with the Pfam database for the presence of three domains of laccases including CuRO_1_LCC_plant (cd13849), CuRO_2_LCC_plant (cd13875), and CuRO_3_LCC_plant (cd13897). *Arabidopsis* laccase protein sequences were aligned as queries against the *Aeluropus Proteome* and genome version 1 (Hashemi-Petroudi et al., 2022) using the local BLASTP and TBLASTN program, respectively. The resulting peptide sequences were verified with the blastP tool in NCBI and the Pfam database for the presence of conserved laccase (TIGR03389). In order to reduce the redundancy, % identity of all sequences was computed with the Decrease Redundancy program (https://web.expasy.org/decrease_redundancy), and any pairs of sequences with more than 99% identity were removed from the analysis. The use of the Decrease Redundancy program resulted in reduction from 21 putative AlLACs to 15 defined protein sequences. The naming of each laccase gene in *Aeluropus* (AlLAC) was based on the closest known orthologue of *Arabidopsis*.

### Phylogenetic analysis

To construct a phylogenetic tree, *AlLAC*s with their orthologous in *Arabidopsis* (TAIR10) and rice (IRGSP-1.0) were analyzed using the multiple sequence alignment Muscle tool of MEGA v. 11 software (Tamura et al., 2021) with the default parameters. A phylogenetic tree was constructed using the maximum-likelihood (ML) method with 1000 bootstrap replicates.

### Motif analysis, physical and chemical properties of laccase protein sequences

The MEME database was used to predict the conserved motifs of AlLAC protein sequences. The number of conserved motifs was adjusted to 10, and other parameters were set as the default. The gene structure of *AlLAC* genes was constructed using Tbtools (Chen et al., 2020) based on GFF format file for exon and intron location information known from Arabidopsis orthologs. The physical and chemical properties, including number of amino acids, molecular weight (kDa), theoretical pI, grand average of hydropathicity (GRAVY), total number of negatively charged residues (Asp + Glu), total number of positively charged residues (Arg + Lys), and instability index of AlLACs, were calculated using the online ExPASy-ProtParam tool (Gasteiger et al., 2005). The subcellular localization of AlLACs were predicted using WoLF PSORT (Horton et al., 2007) based on default settings.

### Prediction of 3D protein structure and pocket analysis of AlLACs

The Phyre2 server was applied to predict the 3D structure of AlLAC proteins (Kelley et al., 2015). Similar structures were analyzed using the Phyre investigator tool to recognize the pocket site related to the binding region.

### Promoter analysis of *AlLAC* genes

PlantCare (Lescot et al., 2002) was used to study *cis*-regulatory elements in the 1000 bp promoter region. The sequence information was retrieved from *Aeluropus* genome version 1 (Hashemi-Petroudi et al., 2022). The identified *cis*-regulatory elements were classified based on their function, and drawn as a graph.

### Protein-protein interaction network

The STRING v11.5 database was used to identify the interactions of AlLAC proteins based on their orthologues in *Arabidopsis*. The first shell of the network was adjusted to ≤ 20 and the second shell was fixed to no more than 10. Gene ontology (GO) enrichment analysis was used to identify the significant (FDR ≤ 0.05) molecular function, biological process, and cellular component terms presented in the LAC-interaction network using the STRING. Cytoscape v3.8.2 (Shannon et al., 2003) was used to construct the LAC-interaction network.

### Plant materials, growth conditions and applied stress

Seeds of Aeluropus were grown at 25 ± 2°C under 16 hours of light and 8 hours of darkness in the greenhouse at Sari Agricultural Sciences and Natural Resources University. After three weeks, the samples were transferred in groups of three to plastic containers, each containing five liters of Hoagland’s nutrient solution under hydroponic culture (Hoagland and Arnon, 1950). After two months, plants of similar size were selected for exposure to stress conditions. For salinity stress, plants were treated gradually by adding 100 mM salt (NaCl) every 48 hours to a final concentration of 600 mM NaCl. For osmotic stress, plants were treated with 20% PEG 6000 of -0.80 MPa. PEG was added to a plastic container and samples (roots and leaves) were collected at different exposure periods of 0 hour (h), as control sample, 6 hours, 48 hours and one week. For cold stress, the plants were exposed to a temperature of 4°C. Sampling of roots and leaves tissues was done 6 h, 48 h and one week after stress exposure, in leaves three biological replicates. The abscisic acid (ABA) treatment was also done by spraying 100 micromolar hormones on the leaves; two leaf and root tissues were sampled at 3, 6, 24 and 48 hours after applying the treatment, in three biological replicates. Three untreated vases were used as controls.

### RNA extraction and cDNA synthesis

Total RNA was extracted from leaf and root tissue of all three biological replicates using the Threezol kit (Threezol, Riragene, Iran). The quantity and quality of the RNA samples were measured by spectrophotometry and 1.5% agarose gel electrophoresis, respectively. *DNase* I treatment (DNase I RNase-free, Thermo Scientific) was used to remove genomic DNA. After combining the RNA of the biological replicates, cDNA synthesis was performed using a kit (Thermo Scientific) according to the company’s instructions, and diluted five times.

### Realtime-qPCR analysis

The expression levels of the target genes were measured with a Bio-Rad CFX96 machine, using The Maxima SYBR Green/ROX qPCR Master Mix kit (Thermo Scientific) in three technical replicates. The cycling profile started at 95°C for 15 seconds, then 40 cycles of 95°C for 15 seconds and 60°C for 60 seconds. At least one negative control (NTC) was considered for each primer. Melting curve analysis of samples and threshold cycle were calculated with CFX software (Bio-Rad). Normalization of gene expression was done with the geometric mean method using specific reference genes of each tissue. Selecting the appropriate internal control gene is very important when determining gene expression, opting for genes that show the least amount of gene expression changes in different stress conditions (either different tissues or sampled at different times). Specific reference genes for leaf tissue were *GTF* and *U2SnRNP*, and specific reference genes for root tissue were *PRS3* and *EF1a* (Hashemi et al., 2016). Five *AlLAC* genes, *AlLAC5*, *AlLAC12.2*, *AlLAC14*, *AlLAC16.1*, and *AlLAC17.1*, were selected based on bioinformatics analyses and primers were designed using AlleleID software (version 7.5, **Table S1**). Quantitative analysis of the data related to the relative expression level of the studied genes was done using the 2^-△△CT^ method (Livak and Schmittgen, 2001).

## Results

In total, 15 LAC proteins in *Aeluropus littoralis* (AlLAC) were identified (**Tables 1**, **S2**). According to their physicochemical properties, the AlLACs encoded proteins ranging from 175 (AlLAC16.1) to 648 aa (AlLAC7.3). The pI values were predicted to be between 5.17 (AlLAC11.1) and 8.93 (AlLAC17.3); eight of the 15 AlLACs showed a pI greater than 7.0 (**Table 1**). Based on the instability index, four of the 15 AlLACs could be introduced as unstable proteins, while the other 11 were predicted to be stable. In addition, most AlLAC proteins (10 of the 15) showed negative values for the GRAVY index, indicating that AlLACs are more hydrophilic. Based on the prediction of the subcellular localization, AlLACs are predominantly located in organelles such as chloroplasts (Chlo.) and vacuoles (Vacu.) (**Table 1**). Overall, AlLACs showed variation based on their physicochemical properties, suggesting that they may have diverse functions.

**Table 1:** Physicochemical properties of AlLAC gene family members in *A.littoralis*.Accession number and sequences (gene, protein, and CDS) of AlLAC gene family members are provided in **Table S2**.

Gene name	Protein length	MW (kDa)	pI	(Asp + Glu)^1^	(Arg + Lys)^2^	instability index	GRAVY	Protein Subcellular Localization Prediction
*AlLAC5*	594	64.72	8.64	39	44	Unstable	-0.051	Mito. Vacu.
*AlLAC6*	317	35.08	7.79	26	27	Stable	-0.329	Vacu.
*AlLAC7.1*	585	63.17	6.99	43	42	Unstable	0.040	Chlo. Mito.
*AlLAC7.2*	593	65.33	6.44	53	46	Stable	-0.129	Chlo. Extr.
*AlLAC7.3*	648	67.65	5.22	47	27	Stable	0.132	Plas.
*AlLAC11.1*	532	57.56	5.17	51	36	Stable	-0.198	Chlo.
*AlLAC11.2*	511	57.23	8.84	44	50	Stable	-0.358	Nucl.
*AlLAC12.1*	560	61.80	5.60	55	39	Stable	-0.106	Vacu.
*AlLAC12.2*	304	32.87	5.51	23	16	Unstable	0.021	Cyto.
*AlLAC14*	579	63.97	6.40	57	51	Stable	-0.235	Mito. Golg.
*AlLAC16.1*	175	19.37	5.85	17	12	Unstable	-0.186	Cyto.
*AlLAC16.2*	550	61.38	8.40	49	52	Stable	-0.214	Vacu.
*AlLAC17.1*	579	62.97	8.47	32	36	Stable	0.073	Chlo.
*AlLAC17.2*	566	61.56	8.04	35	37	Stable	0.047	Extr. Cyto.
*AlLAC17.3*	569	62.40	8.93	39	46	Stable	-0.086	Chlo.

1: Total number of negatively charged residues (Asp + Glu), 2: Total number of positively charged residues (Arg + Lys).

### Phylogenetic relationships of LAC gene family

**Figure 1:**
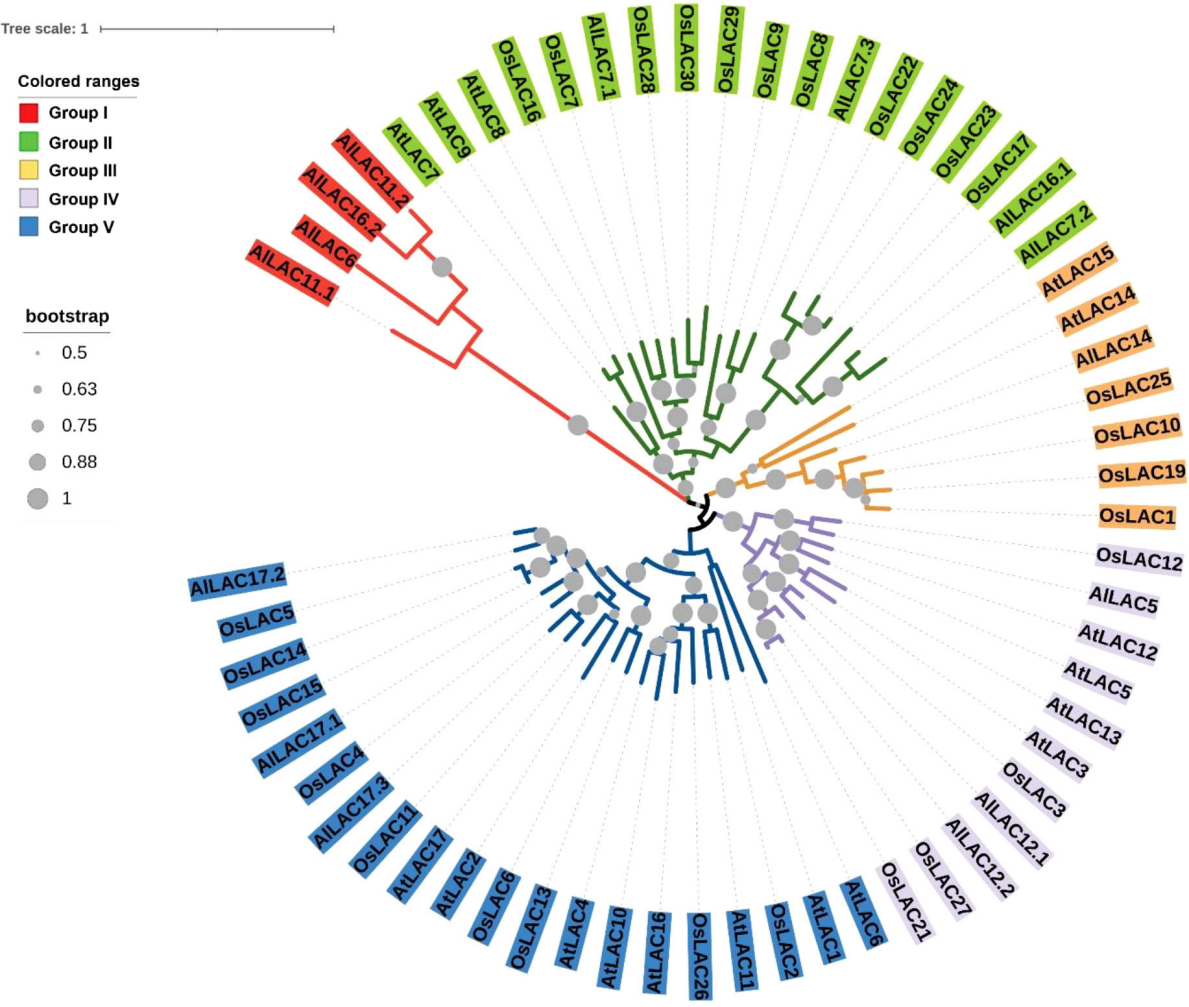
Phylogenetic tree of LAC gene members in *Arabidopsis* (AtLAC), rice (OsLAC), and *A.littoralis* (AlLAC).

**Figure 2:**
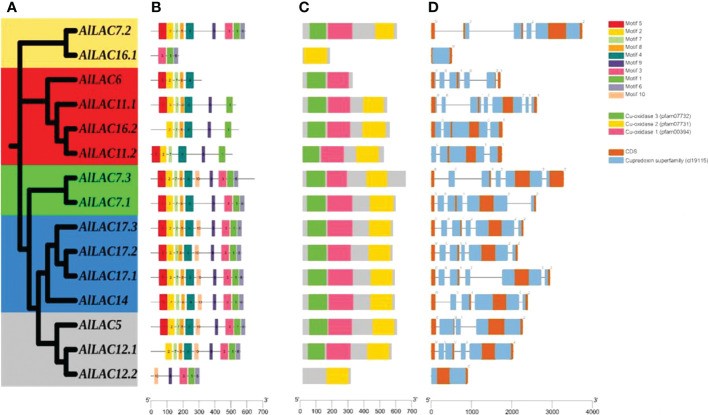
Phylogenetic tree of AlLAC proteins **(A)**, conserved motif distribution of AlLAC proteins **(B)**, conserved domains of AlLAC proteins **(C)**, and gene structure of *AlLAC* genes **(D)**. The logo and sequence of conserved motifs are provided in **Table S3**.

The phylogenetic tree for members of the LAC gene family in *Arabidopsis* (*AtLACs*), rice (*OsLACs*), and *A. littoralis* (*AlLACs*) separated LACs into five main groups (**Figure 1**). Furthermore, *AlLAC*s were more similar to their orthologs in rice, suggesting that a close evolutionary process may have occurred in this gene family in rice and *A. littoralis*. Moreover, it seems that the diversity of LAC family has more occurred after derivation of monocots and dicots. *AlLAC*s from group I, including *AlLAC11.1*, *AlLAC6*, *AlLAC16.2*, and *AlLAC11.2* showed a greater genetic distance than other members, suggesting that these members may have a higher potential for further molecular functional investigation. In addition, the gene structure, conserved motifs, and encoded domain distribution of *AlLAC*s were analyzed (**Figure 2** and **Table S3**). Ten conserved motifs and three encoded domains were identified in *AlLAC*s. Motif 4 was in the Cu-oxidase 1 domain region, while motifs 6, 1, and 3 were predicted in Cu-oxidase 2 domain, and motifs 5 and 2 were located in Cu-oxidase 3 domain (**Figure 2**). Some of the conserved motifs (motif 9 and motif 10) were identified in the extra-domain region that can be used to identify the *AlLAC*s. Among the various gene structures observed in *AlLAC*s, the maximum exon/intron number was observed in *AlLAC11.1*.

### Structural analysis of AlLAC proteins

**Figure 3:**
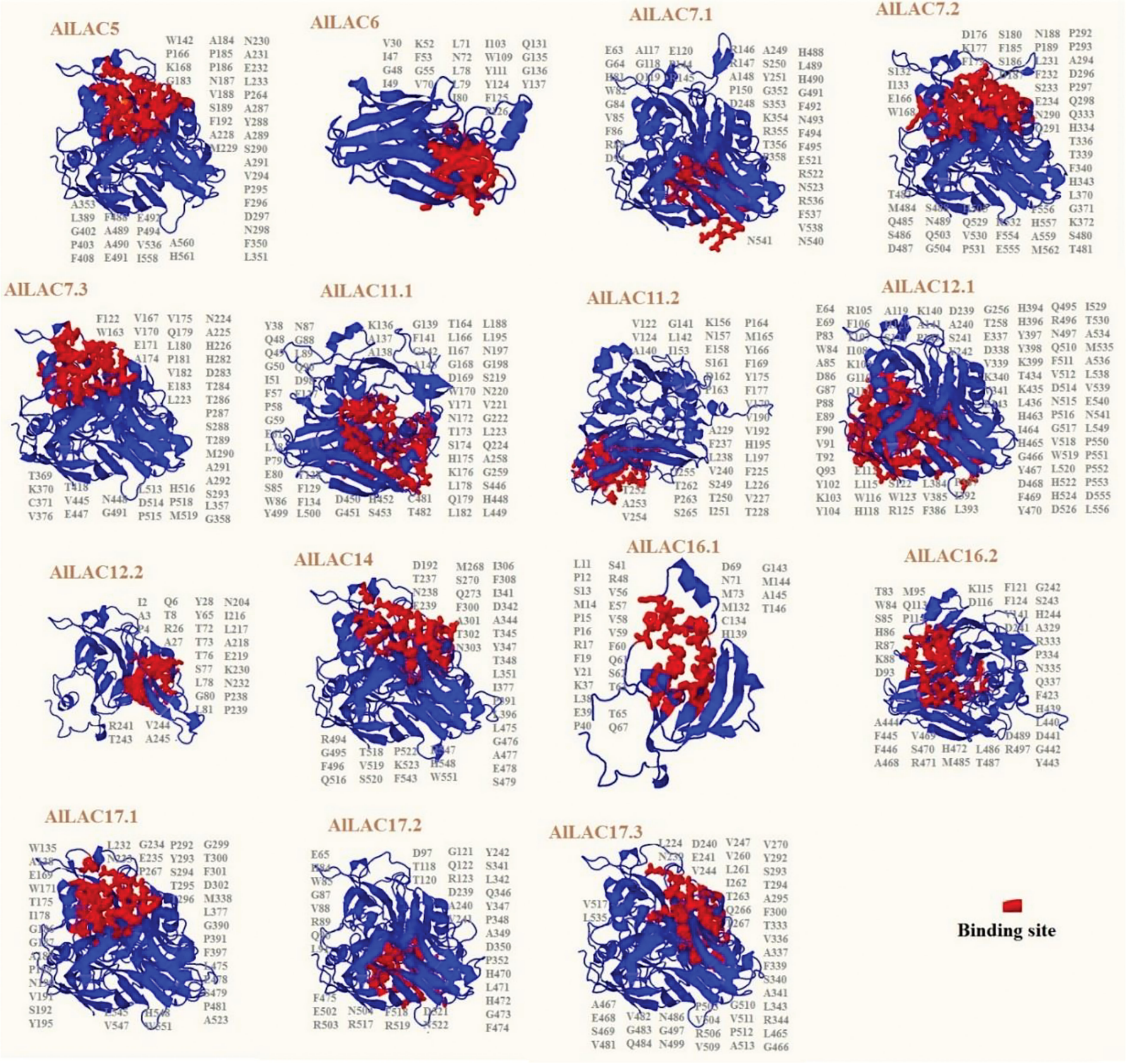
Three-dimensional docking analysis of AlLACs. The binding residues are shown on protein structure.

**Figure 4:**
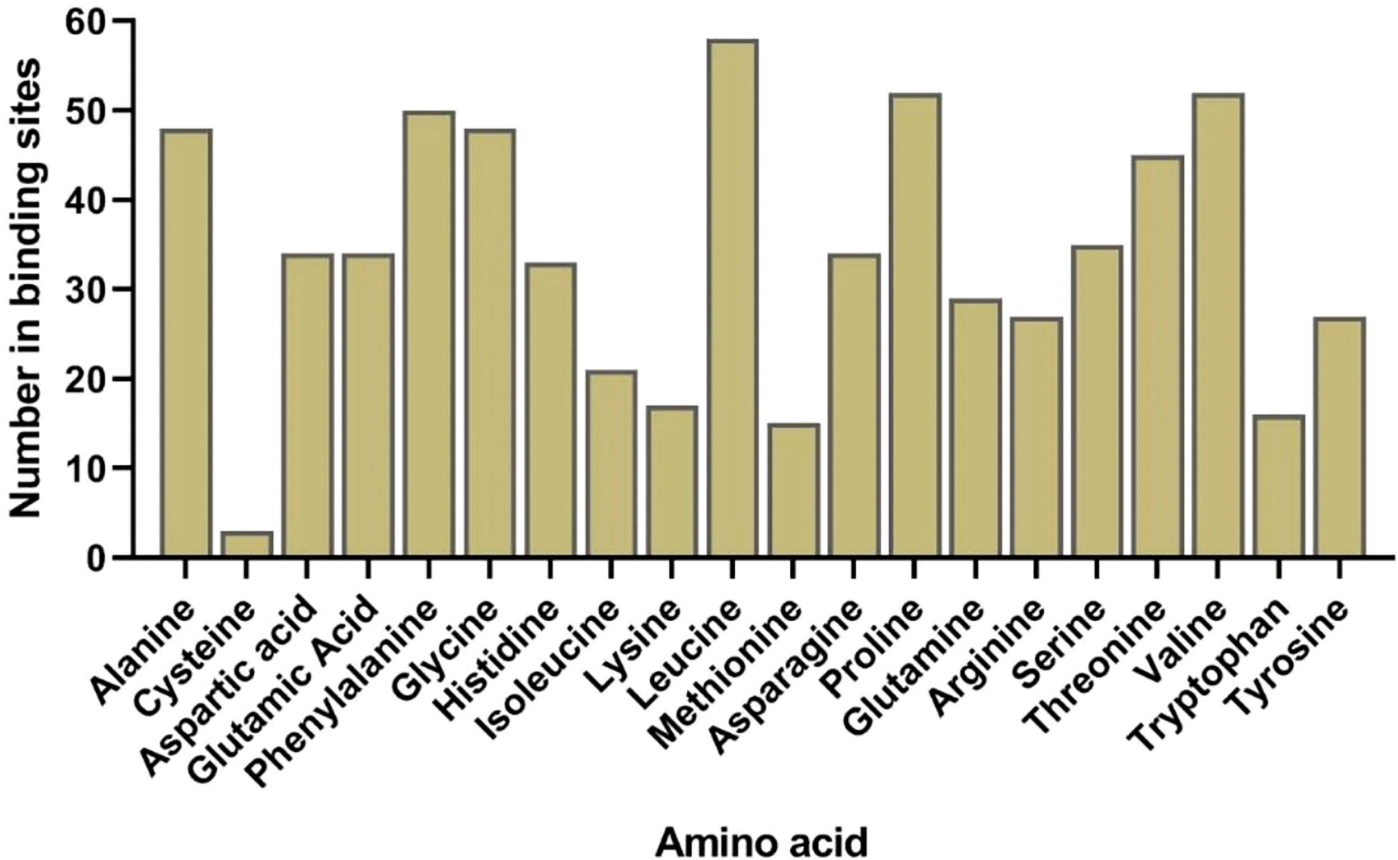
Frequency of each amino acid in ligand-binding sites in all studied AlLAC proteins.

The predicted 3D structure and binding sites of AlLACs revealed diverse structures among members (**Figure 3**). Moreover, leucine (L), proline (P), valine (V), phenylalanine (F), glycine (G), and alanine (A) were frequently predicted in the binding site region of AlLACs (**Figure 4**). The residues detected in the pocket sites of all AlLACs demonstrate the key positions of the important amino acids likely related to their function and interaction points.

### Protein-protein interaction of LACs

**Figure 5:**
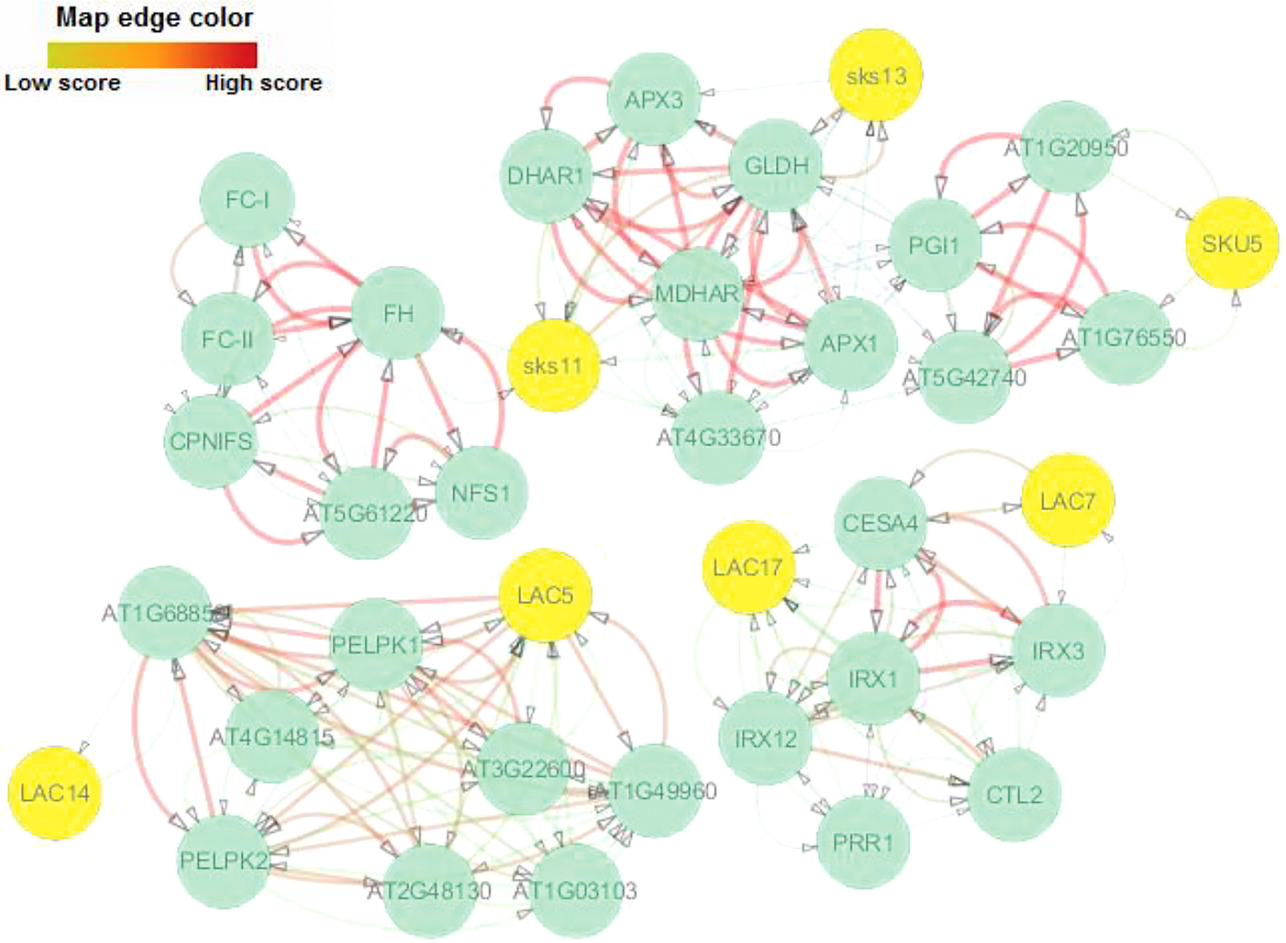
Protein–protein interaction network of AlLACs using STRING server v11 based on *Arabidopsis* interactome data.

The interaction network for AlLAC proteins was drawn based on their orthologs in the model plant, *Arabidopsis*. Sks11, sks13, and SKU5 proteins, which have oxidoreductase activity, were the most similar to AlLAC11.2, AlLAC16.2, and AlLAC6 proteins, respectively (**Figure 5**). There were no direct interactions between the laccases themselves. Three interaction groups were observed: the first group contained Sks11 (AT3G13390), sks13 (AT3G13400), and SKU5 (AT4G12420); the second group contained LAC7 and LAC17; and the third group contained LAC5 and LAC6. LAC5 showed the most and strongest interactions compared to the other laccases. Gene ontology (GO) enrichment analysis revealed several biological process terms; iron incorporation into metallo-sulfur cluster, lignin catabolism, regulation of symbiotic processes, plant-type primary cell wall biogenesis, and L-ascorbic acid biosynthesis were significantly linked with the LAC-interaction network (**Table S4**). In addition, molecular function terms including cysteine desulfurase activity, glucose-6-phosphate isomerase activity, ferrochelatase activity, copper ion binding, oxidoreductase activity, cellulose synthase (udp-forming) activity, and antioxidant activity were significantly associated with the LAC-interaction network (**Table S4**).

### Promoter analysis

**Figure 6:**
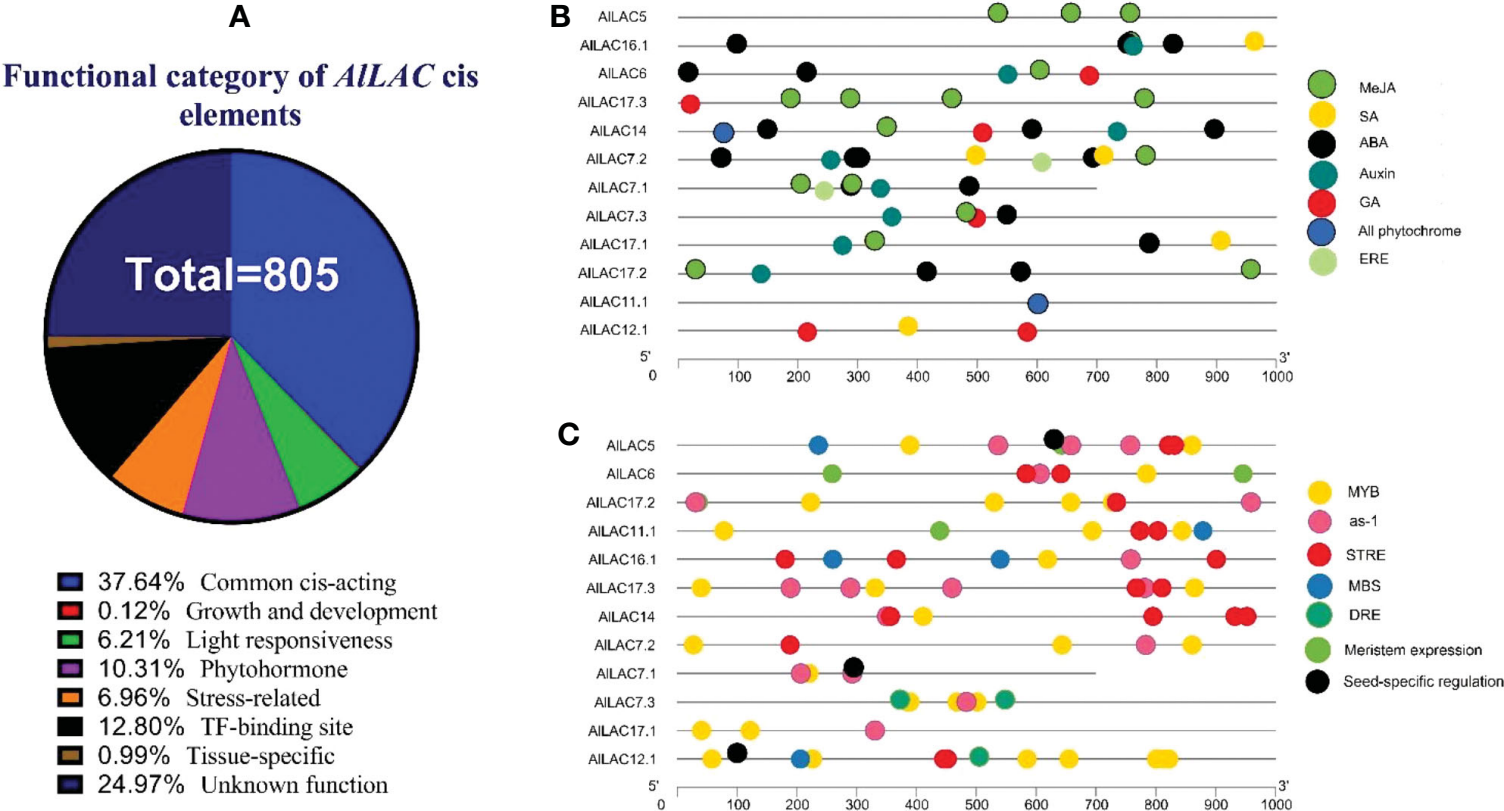
*Cis*-regulatory distribution in the promoter region of *AlLAC* genes. Percentage of *cis*-regulatory elements based on their functions **(A)**, distribution of *cis*-regulatory elements related to phytohormone **(B)**, and distribution of *cis*-regulatory elements related to growth and development, TF binding site, and stress responsiveness **(C)**. More details of *Cis*-regulatory distribution in the promoter region of *AlLAC* genes are provided in **Table S5**.

The upstream region of *AlLAC* genes was analyzed to detect putative *cis*-regulatory elements. The *cis*-regulatory elements that are involved with stress, growth and development, phytohormones, and transcription factor (TF) site were observed in the promoter region (**Figure 6**). The common and unknown function of *cis*-regulatory elements were frequently observed upstream of *AlLAC*s (**Figure 6A**). *Cis*-regulatory elements to methyl jasmonate (MeJA) and ABA responsiveness (ABRE) were frequently detected in the promoter region (**Figure 6B**). Moreover, acting elements related to ethylene (ERE), salicylic acid (SA), gibberellic acid (GA), and auxin responsiveness were also observed in the promoter region (**Figure 6B**), as were stress-responsive MYB elements such as as-1, an acting element involved in oxidative stress-responsive, and STRE (stress-controlled transcription factors) elements (**Figure 6C**). Dehydration-responsive element (DRE) and MBS (MYB binding site involved in drought-inducibility) elements were also observed upstream of *AlLAC*s (**Figure 6C**). These results suggest that laccases probably cooperate with factors involved in stress response and are probably present in stress-dependent signaling pathways.

### Expression in response to ABA application

**Figure 7:**
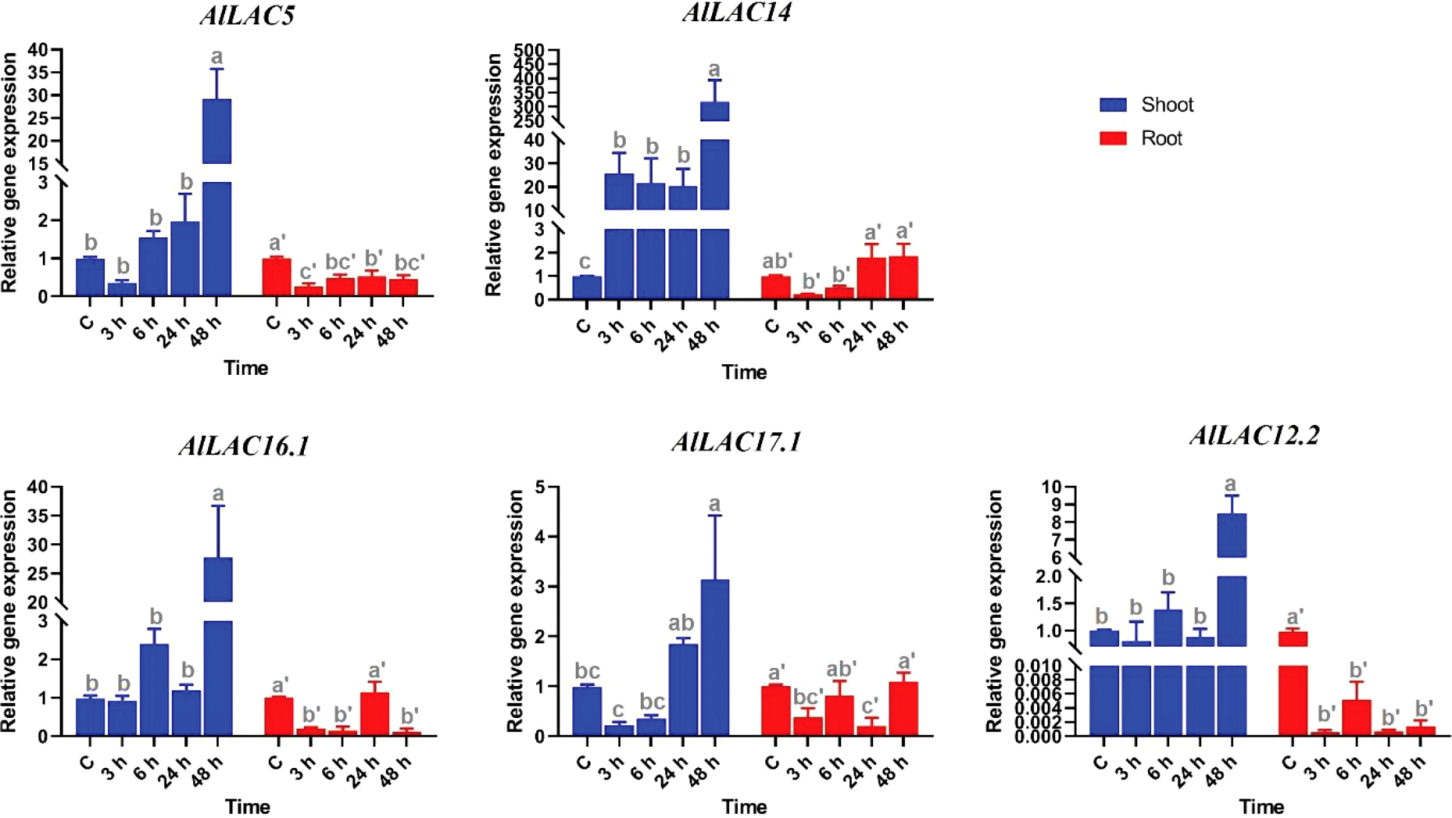
Expression profile of *AlLAC* genes under ABA treatments. Different letters above each bar indicate a significant difference (p < 0.05) based on Tukey’s range test.

The expression levels of five candidate *AlLAC* genes (*AlLAC5*, *AlLAC14*, *AlLAC16.1*, *AlLAC17.1*, and *AlLAC12.2*) were investigated under ABA application in shoot and root tissues of *A. littoralis* (**Figure 7**). The *AlLAC* genes for real time RT-PCR analysis were selected on their mRNA abundance and salt stress inducibility reported in (Younesi-Melerdi et al., 2020). Based on their expression profile, all selected genes illustrated tissue-specific expression, and they were highly expressed in shoot tissues. Interestingly, all studied *AlLAC*s were sharply upregulated after 48 hours of ABA application in shoot tissues, while *AlLAC14* was upregulated at all-time points of ABA application in shoot tissues. Besides, all *AlLACs* showed a down-regulation after 3 h of ABA application in root tissues. Overall, ABA may indirectly affect the expression of *AlLAC*s in the shoot.

### Expression profile of *AlLAC* genes under abiotic stresses

**Figure 8:**
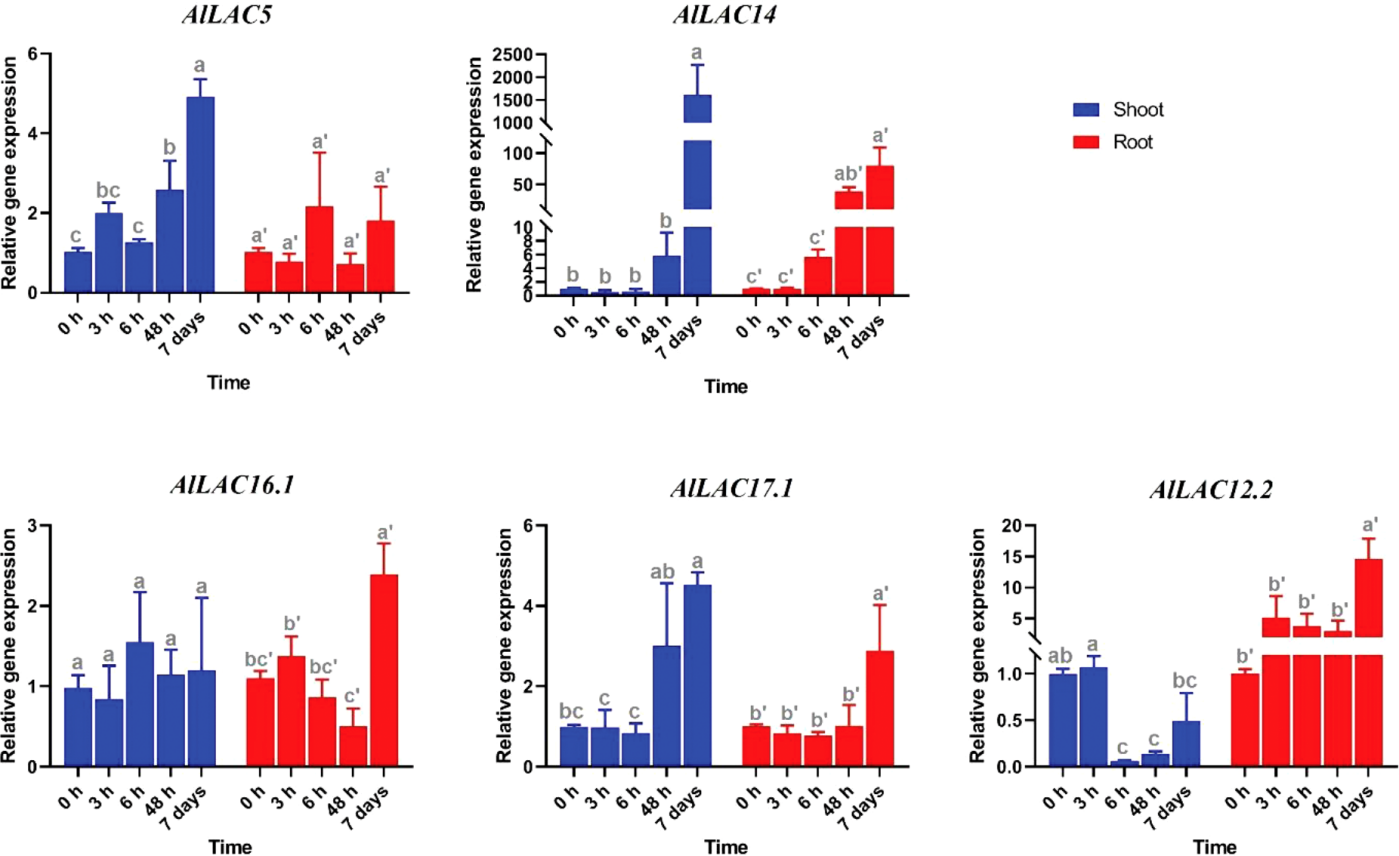
Expression profile of *AlLAC* genes in response to cold stress. Different letters above each bar indicate a significant difference (p < 0.05) based on Tukey’s range test.

**Figure 9:**
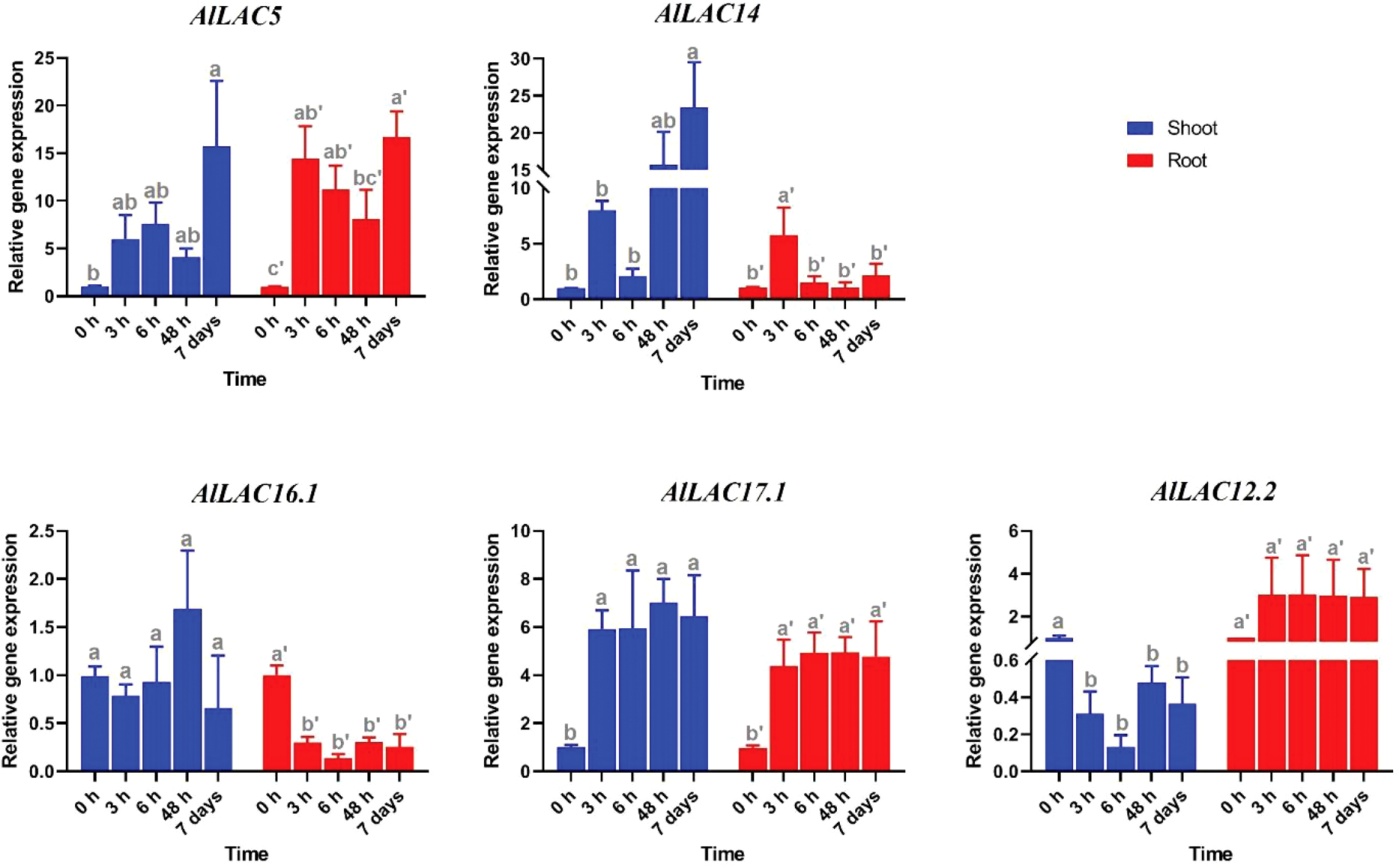
Expression profile of *AlLAC* genes in response to salt stress. Different letters above each bar indicate a significant difference (p < 0.05) based on Tukey’s range test.

**Figure 10:**
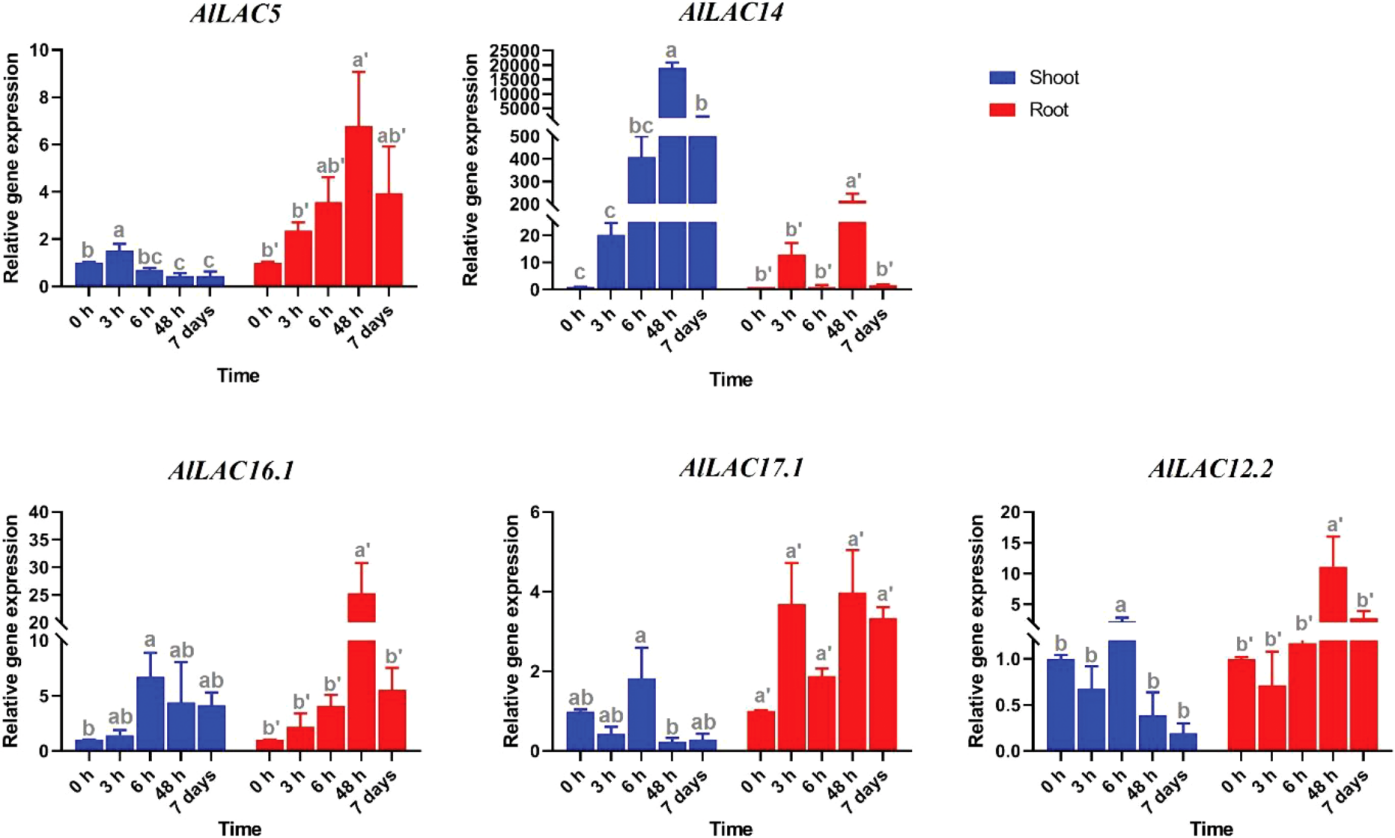
Expression profile of *AlLAC* genes in response to osmotic stress (PEG application). Different letters above each bar indicate a significant difference (p < 0.05) based on Tukey’s range test.

Expression patterns of five selected *AlLAC* genes in response to abiotic stresses – cold, osmotic (using PEG application), and salt stress – were evaluated in shoot and root tissues (**Figures 8**-**10**). In response to cold stress, *AlLAC5* was upregulated after seven days in shoot tissues, while *AlLAC14* was highly induced after 48 hours and seven days in both shoot and root tissues (**Figure 8**). *AlLAC16.1* and *AlLAC17.1* were less induced in response to cold stress, and *AlLAC12.2* showed upregulation at all studied time points, especially after seven days, in root tissues. In response to salt stress, *AlLAC* genes showed diverse expression patterns. For instance, *AlLAC5, AlLAC17.1*, and *AlLAC12.2* were upregulated in roots, while *AlLAC14* showed a high upregulation in the shoot (**Figure 9**). Furthermore, *AlLAC5*, and *AlLAC17.1* were upregulated in both root and shoot, suggesting that these genes are directly associated with response to salt stress. In addition, *AlLAC16.1* showed a down-regulation in root tissues at all-time points in response to salinity (**Figure 9**). The expression profile of *AlLAC*s was analyzed under PEG application for induction of osmotic stress. Interestingly, all studied genes showed an upregulation in root tissues, and high expression was recorded after 48 h (**Figure 10**). In shoot tissues, *AlLAC14* was sharply induced after 48 hours in response to drought stress. Overall, *AlLAC14* appears to be more expressed in shoot tissues, and *AlLAC*s are more involved in the late response of *A. littoralis* to abiotic stress.

## Discussion

Laccases have important roles such as lignin polymerization in plants, and also contribute to resistance to environmental stresses (Bao et al., 1993; Sato et al., 2001; Berthet et al., 2011). Enzymes of this gene family moved into focus due to their ability to catalyze steps in bio-economy (Arregui et al., 2019; Agustin et al., 2021) and their utilization as biosensors (Lee et al., 2018). Thus far, laccases have not been studied in *Aeluropus littoralis*, a model plant that is highly resistant to salinity stress (Saad et al., 2011; Hashemi et al., 2016). *A. littoralis* has a high potential in the field of resistance to abiotic stresses and serves to identify and isolate new stress-adapted genes. In the current study, 15 *AlLAC*s were identified from the *A. littoralis* genome. Previous studies have identified 30 LACs from rice (Liu et al., 2017), 40 LACs from the pear genome (Lu et al., 2021), 52 LACs from the *Setaria viridis* genome (Simões et al., 2020), 54 LACs from the *Eucalyptus grandis* genome (Arcuri et al., 2020), 29 LACs from the *Brachypodium distachyon* genome (Wang et al., 2015b), 17 LACs from the *Arabidopsis thaliana* genome (Turlapati et al., 2011), and *27* LACs from the *Sorghum bicolor* genome (Wang et al., 2017). The lower number of laccase genes found here suggests that the laccase gene family is probably less extended in *A. littoralis* during evolutionary processes. Based on their physicochemical characteristics and gene structure, *AlLAC*s were diverse, indicating that the members of this gene family are involved in various cellular processes (Ahmadizadeh et al., 2020; Heidari et al., 2021a). Intron/exon number was also diverse among *AlLAC*s. It was stated that the number of exons/introns can affect the expression levels; genes with fewer exons can be rapidly activated (Jeffares et al., 2008; Koralewski and Krutovsky, 2011; Iñiguez and Hernández, 2017; Heidari et al., 2022). According to phylogenetic analysis, LAC gene family members from *A. littoralis*, *Arabidopsis*, and rice were grouped into five classes, and a close evolutionary process was observed between *AlLAC*s and their orthologues in rice. This finding suggests that mutations have occurred in coding sequence regions after the divergence between dicots and monocots (Faraji et al., 2021a; Musavizadeh et al., 2021). In addition, the distribution of conserved motifs might be associated with the diversity and function of genes from a family (Faraji et al., 2021b). Some conserved motifs were observed in the extra-domain site – these regions can be used to identify *AlLAC*s and their role of these proteins in stress response.

AlLACs also showed diverse 3D structures. Leucine, proline, valine, phenylalanine, glycine, and alanine were identified as the amino acids that affect the interaction and function of AlLACs. The interaction network for AlLAC proteins based on their orthologues in the model plant, *Arabidopsis*, suggested that laccases are probably involved in diverse cellular processes related to cellulose synthase activity and oxidoreductase activity, including iron incorporation into metallo-sulfur cluster, lignin catabolism, regulation of symbiotic processes, plant-type primary cell wall biogenesis, and L-ascorbic acid biosynthesis. Previous studies have also stated that laccases are associated with processes related to plant cell wall components (Cesarino et al., 2013; Liu et al., 2017). Notably, the weak interaction between the laccases indicates that they likely work in independent pathways.

Several classes of *cis*-acting elements related to response to hormones, light, abiotic and biotic stresses, growth and development processes were identified upstream of *AlLAC* genes, suggesting that the members of this gene family have multifunctional roles in *A. littoralis* (Ahmadizadeh and Heidari, 2014; Rezaee et al., 2020). We investigated the expression profile of five *AlLAC* genes in response to ABA and PEG application, cold, and salt stress using qRT-PCR. Notably, *AlLAC*s showed differential expression patterns in shoot and root tissues, indicating that these genes have different functions. Our findings demonstrated that *AlLAC14* is more induced in shoot tissues after 48 hours of exposure to stresses, however further molecular functional studies of this gene are recommended. In addition, the selected *AlLAC* genes were upregulated in response to application of ABA hormone, a stress-dependent regulator that many signaling pathways use in response to adverse environmental conditions (Heidari et al., 2021b). The induction of *AlLAC* genes after 48 hours of ABA application raises that these genes probably have some interaction with signaling pathway related to ABA. Furthermore, the selected *AlLAC* genes illustrated diverse expression in response to abiotic stresses, including cold, salt, and osmotic stress. Interestingly, these *AlLAC*s were induced after 48 hours of exposure to stress conditions, revealing that *AlLAC*s are associated with late response pathways of *A. littoralis*. Several studies have mentioned the role of laccases in the process of lignification (Sato et al., 2001; Berthet et al., 2011). Laccases involves in lignin polymerization and can affects the lignin synthesis (Berthet et al., 2011). Lignin is a component of plant cell walls that plays an important role in increasing the resistance and strength of plants. Besides, it was reported that LACs can be regulated by transcription factor MYB and be induced by abiotic stresses (Xu et al., 2022). Our results suggest that laccases are involved in response to adverse conditions from direct and indirect pathways in *A. littoralis*.

## Conclusion

The present study identified 15 *AlLAC*s in the *Aeluropus littoralis* genome, which formed five groups based on phylogenetic analysis. *AlLAC*s showed high diversity in structure and physicochemical properties, suggesting that these gene family members were influenced by evolutionary pressure. Various *cis*-regulatory elements were observed upstream of *AlLAC*s, revealing that *AlLAC*s are involved in different cell signaling pathways. Moreover, *AlLAC*s showed tissue-specific expression, suggesting that *AlLAC* genes might be associated with growth and development processes. All of the selected *AlLAC* genes were induced in response to salt, osmotic, and cold stress, indicating that they can play a role in increasing the tolerance to adverse conditions.

## Data availability statement

The datasets presented in this study can be found in online repositories. The names of the repository/repositories and accession number(s) can be found in the article/**Supplementary Material**.

## Author contributions

SH and MA performed the experiments, SH MA and MK analyzed the data and wrote the manuscript. PH contributed to the data analysis and preparation of the manuscript. All authors contributed to the article and approved the submitted version.

## Supplementary material

Supplementary Table 1 List of *AlLAC* genes primers used in real-time PCR.

Supplementary Table 2 Accession number and sequences (gene, protein, and CDS) of *AlLAC* genes.

Supplementary Table 3 Sequence and logo of conserved motifs distributed in AlLAC proteins.

Supplementary Table 4 Gene ontology (GO) enrichment of AlLAC-interaction network.

Supplementary Table 5 Functional category of AlLAC promoter element.
