# Supplementary material for: Genome-wide analysis of the laccase (LAC) gene family in Aeluropus littoralis: A focus on identification, evolution and expression patterns in response to abiotic stresses and ABA treatment
Source: Front Plant Sci. 2023 Mar 1;14:1112354. doi: 10.3389/fpls.2023.1112354 (PMC10014554; doi:10.3389/fpls.2023.1112354)
Supplement: Supplementary Table 1 — List of AlLAC genes primers used in real-time PCR. [file Table_1.docx]

| Gene ID | Sequence | Length | Tm | GC % | Amplicon length | Amplicon Tm | Tm anealing |
| --- | --- | --- | --- | --- | --- | --- | --- |
| AlLAC14 | GCCTACTACCGCTCCATC | 18 | 63.2 | 61.1 | 140 | 85.9 | 64.1 |
|  | TACTCCAGAACCTTCACCTTG | 21 | 63.3 | 47.6 |  |  |  |
| AlLAC17.1 | TCTGCTCTTCCTTGCTCTC | 19 | 63.0 | 52.6 | 149 | 83.2 | 62.1 |
|  | TTAACCGTGATGATGCTCTTG | 21 | 62.5 | 42.9 |  |  |  |
| AlLAC5 | TCCTCCTCTGCTCTCCTT | 18 | 62.9 | 55.6 | 105 | 83.5 | 62.4 |
|  | CTTCACTGGCGTCTCCTT | 18 | 63.2 | 55.6 |  |  |  |
| AlLAC12.2 | CAGAAGCAAGCCTGTTGTC | 19 | 63.2 | 52.6 | 166 | 86.3 | 64.4 |
|  | AGCGGAGCAGTTGAAGAG | 18 | 63.6 | 55.6 |  |  |  |
| AlLAC16.1 | CTTGCTCAGGGTCTTGGTA | 19 | 62.9 | 52.6 | 87 | 78.8 | 59.1 |
|  | TTGTACGGTGTTCTTCAGTAGT | 22 | 63.2 | 40.9 |  |  |  |
